# Zebrafish embryos hatch early in response to chemical and mechanical indicators of predation risk, resulting in underdeveloped swimming ability of hatchling larvae

**DOI:** 10.1242/bio.059229

**Published:** 2022-11-29

**Authors:** Brian D. Wisenden, Daniel C. Paulson, Megan Orr

**Affiliations:** ^1^ Biosciences Department, Minnesota State University Moorhead, Moorhead, MN 56563, USA; ^2^ Department of Statistics, North Dakota State University, Fargo, ND 58108, USA

**Keywords:** Environmentally induced hatching, Chemical alarm cues, Mechanical disturbance, Zebrafish, Hatch time, Swimming speed, Predation risk

## Abstract

Plasticity in hatching time allows embryos to maximize fitness by balancing the benefits and costs of remaining bound within the chorion against the benefits and costs of emerging as a free-swimming larva. Here, in the first experiment, we exposed zebrafish (*Danio rerio*) embryos to either chemical cues from crushed embryos (simulating egg predation) or to blank water control. Embryos exposed to alarm cues hatched sooner, and had shorter body lengths and underdeveloped fins, relative to larvae from the water treatment. Burst swimming speed was significantly slower for larvae that hatched from the alarm cue treatment than for larvae from the water treatment. In a second 2×2 experiment, we exposed zebrafish embryos to either chemical alarm cues from conspecific embryos, mechanical disturbance (magnetic stir bar) to simulate a predator probing the substrate for developing embryos, both chemical and mechanical indicators of risk, or neither (control). We found similar effects in terms of earlier time to hatch at an earlier stage of development and poorer swimming performance of hatchling larvae. In the second experiment, these effects occurred in response to mechanical disturbance with or without the presence of chemical alarm cues. Alarm cues alone produced no effects in the second experiment. Taken together, these data indicate that zebrafish embryos demonstrate a facultative trade-off between risk of predation acting on two stages of their life history.

## Teaser

**Summary:** Zebrafish embryos can hatch early if they sense chemical or mechanical indicators of predation risk, but when they hatch early they are poorly developed and more vulnerable to predators as larvae.

## INTRODUCTION

Predation is often the final arbiter of natural selection and therefore predation, and/or the risk of predation, exerts strong effects on all aspects of life history, ecology and evolution. Predator-prey interactions are an evolutionary arms race between foraging efficiency of predators versus evasion and avoidance behaviors by prey (Lima and Dill, 1990). Prey organisms use a wide variety of sensory modalities to detect the presence of predation risk. In aquatic habitats, chemical cues released during predator-prey interactions provide information about the identity of the predator and about the exigency of risk (Kats and Dill, 1998; Ferrari et al., 2010; Wisenden, 2015). For example, prey attend to signature odors of predators (kairomones), and chemical information released from disturbed or startled conspecifics. A large literature has been devoted to the role of alarm cues, analogous to ‘death cries’, which comprise water-soluble compounds released from prey tissues damaged by predator attack. Finally, prey detect chemical indicators of predation risk from a predator's diet released from the predator's feces (Ferrari et al., 2010). Prey that detect these cues alter their behavior in ways that reduce the probability of predation. The most commonly studied behavioral responses are area avoidance, reduction in activity, increased shoal cohesion, and increased time out of the water column and/or under cover (Ferrari et al., 2010; Wisenden, 2015). Prey species may also exhibit morphological responses to chemical cues. For example, some fish deepen their body to thwart gape-limited predators (e.g. Brönmark and Miner, 1992; Meuthen et al., 2019; Díaz-Gil et al., 2020), tadpoles can alter tail shape for different swimming responses (Relyea, 2004; Laurila et al., 2001), cladocerans grow helmets and spines to deter predation (Tollrian and Harvell, 1999), and gastropods alter shell shape (Bourdeau et al., 2015; Dalesman et al., 2015).

Behavioral and morphological responses are not limited to juveniles and adults. Embryos can alter morphological and behavioral phenotype by delaying time to hatch to avoid risk of predation on hatchlings (Sih and Moore, 1993; Moore et al., 1996; Laurila et al., 2002) or accelerate time to hatch to reduce exposure to predators of embryos (Sih and Kats, 1994; Warkentin, 1995; Chivers et al., 2001; Mirza et al., 2001; Kusch and Chivers, 2004). Moreover, embryos can acquire predator recognition by attending to external cues before hatching (Mathis et al., 2008; Ferrari and Chivers, 2009; Nelson et al., 2013; Horn et al., 2019; Horn and Chivers, 2021a).

Early hatching may present a trade-off in that hatching prematurely confers the fitness benefit of avoiding an imminent attack by a predator of embryos, but at a cost of hatching at an earlier stage of development, which results in relatively weak antipredator competence at hatch (Warkentin, 1995, 2011; Kusch and Chivers, 2004; Capellán and Nicieza, 2007). For example, vibrational stimuli created by snakes attacking arboreal clutches of embryonic red-eye tree frogs cue embryos to hatch prematurely (Warkentin, 1995, 2005, 2006) and drop from riparian vegetation into the water below. However, embryos that hatch early to escape from snakes are smaller and more easily caught by aquatic predators that await newly hatched tadpoles (Warkentin, 1995, 1999a,b; Touchon et al., 2013; Willink et al., 2014). Similarly, fathead minnow embryos hatch early, at a smaller size, when exposed to the odor of crayfish fed a diet of minnow embryos (Kusch and Chivers, 2004).

**Fig. 1.**
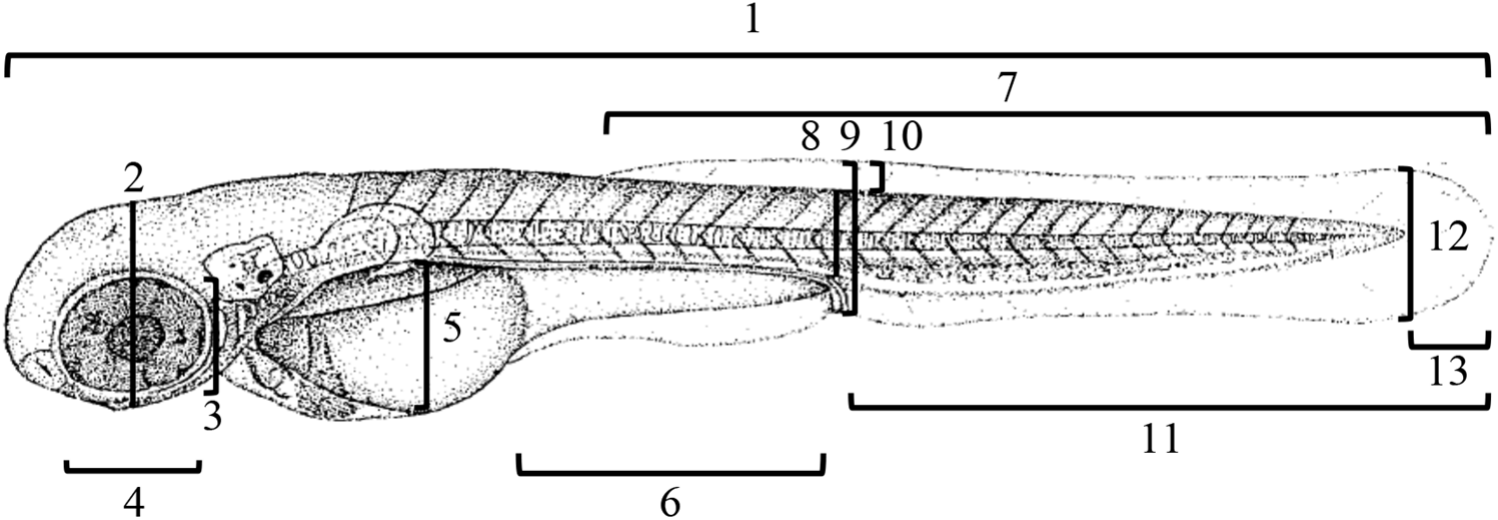
**Morphological measurements of newly hatched zebrafish larvae.** 1, total length; 2, head depth; 3, eye height; 4, eye length; 5, yolk sac height; 6, body cavity length; 7, dorsal fin length; 8, trunk height; 9, total trunk height; 10, dorsal fin height; 11, anal fin length; 12, caudal fin height; 13, caudal fin length. Line drawing from Kimmel et al. (1995). This image is not published under the terms of the CC-BY license of this article. For permission to reuse, please see Kimmel et al. (1995).

Here, we exposed zebrafish embryos to indicators of predation in two sensory modalities: chemosensory indicators of risk in the form of chemical alarm cues derived from crushed conspecific embryos and/or a standardized mechanical disturbance to simulate vibrational cues created by an egg predator probing for embryos in the substrate. Zebrafish are known to have a plastic hatching period (Kimmel et al., 1995) and, as adults, respond behaviorally to conspecific alarm cue (e.g. Waldman, 1982; Hall and Suboski, 1995; Korpi and Wisenden, 2001; Speedie and Gerlai, 2008; Barkhymer et al., 2018). We measured time to hatch, larval morphology at hatch (Fig. 1) and swimming performance at hatch. We predicted that embryos exposed to either chemical (alarm cue) or mechanical (stir bar) indicators of risk would hatch relatively early, at an earlier stage of development, and consequently would show weaker swimming ability compared to control embryos.

## RESULTS

### Experiment #1: Effect of chemical indicators of predation risk

**Fig. 2.**
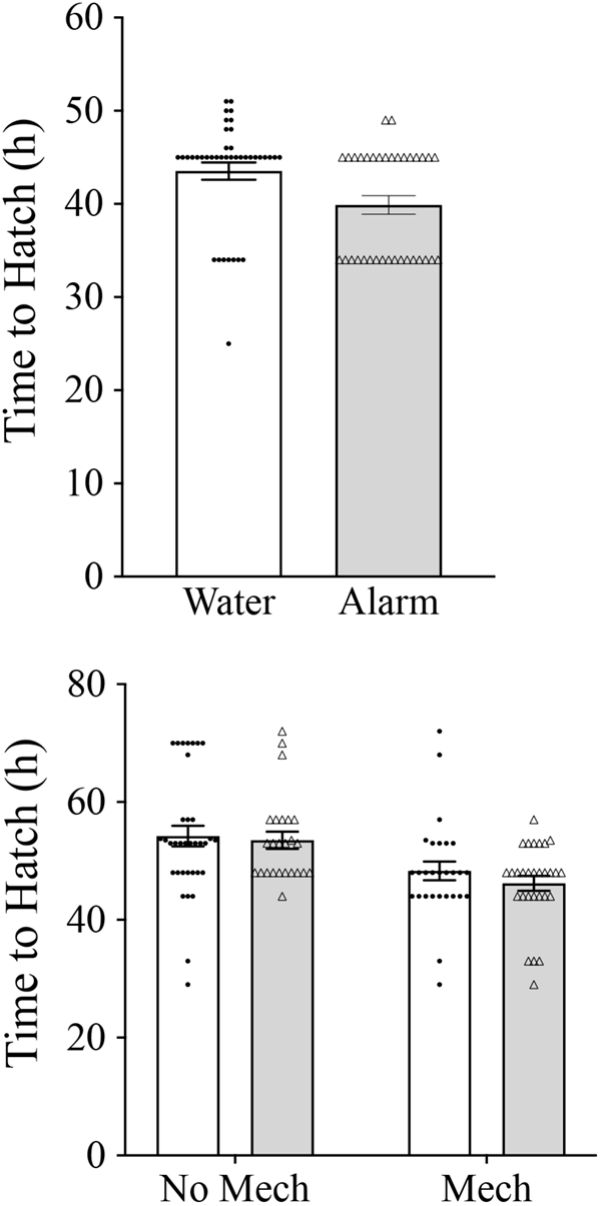
**Effects of chemical alarm and mechanical cues on time to hatch.** Chemical alarm cues reduced time to hatch in the first experiment (ANOVA *F*_1,45_=6.09, *P=*0.017). In the second experiment, mechanical cues reduced time to hatch (ANOVA *F*_1,66_=12.51, *P=*0.001), but there was no effect of chemical alarm cue (ANOVA *F*_1,66_=0.19, *P=*0.665) or its interaction with mechanical disturbance (ANOVA *F*_1,66_=0.05, *P=*0.831). Mean±s.e. time (h) to hatch for embryos exposed to blank water (open bars, *n*=40) or chemical alarm cues (shaded bars, *n*=35) of conspecific embryos in the first experiment (top), and for embryos exposed to chemical and/or mechanical indicators of predation risk in the second experiment (sample sizes: control, *n*=35; mechanical disturbance only, *n*=28; chemical alarm cues only, *n*=25; both chemical and mechanical indicators of risk, *n*=29) (bottom).

**Fig. 3.**
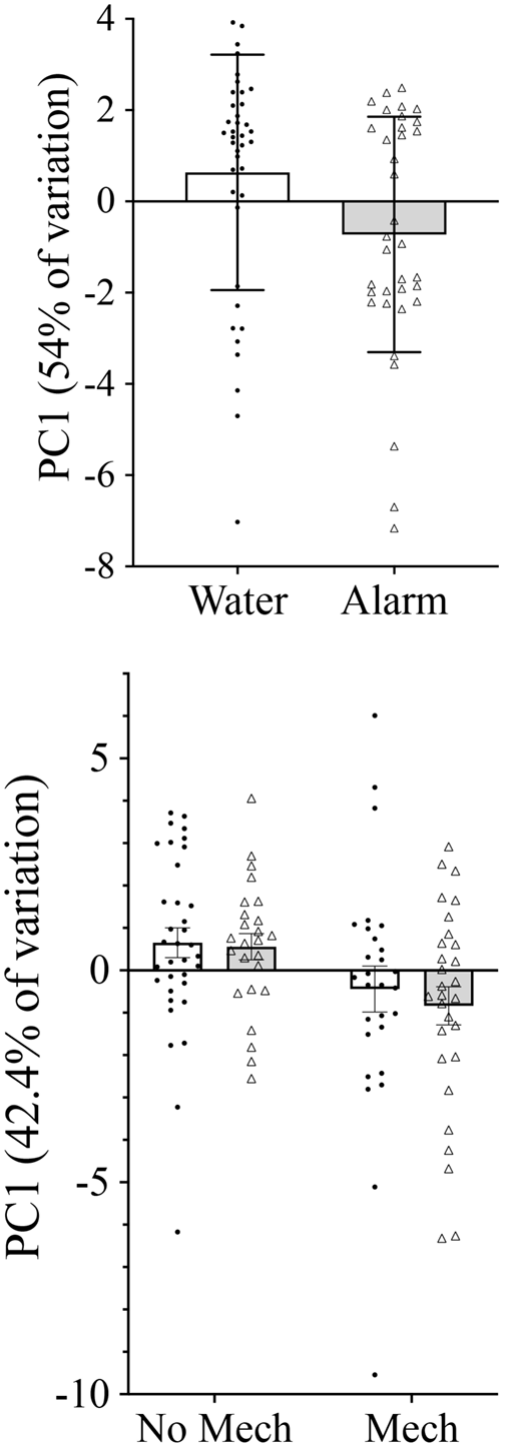
**Effect of chemical alarm and mechanical cues on larval morphology.** Chemical alarm cues caused embryos to hatch at earlier stage of development in the first experiment (ANOVA *F*_1,45_=5.34, *P=*0.026). In the second experiment, only mechanical cues affected developmental stage at hatch (mechanical cues: ANOVA *F*_1,66_=6.91, *P=*0.011; chemical alarm cues: *F*_1,66_=0.03, *P=*0.868; chemical cues * mechanical cues: *F*_1,66_=15, *P=*0.697). Mean±s.e. principal component axis score (PC1) of morphological traits of embryos exposed to blank water (open bars, *n*=40) or chemical alarm cues (shaded bars, *n*=35) of conspecific embryos in the first experiment (top), and chemical and/or mechanical indicators of predation risk in the second experiment (sample sizes: control, *n*=35; mechanical disturbance only, *n*=28; chemical alarm cues only, *n*=25; both chemical and mechanical indicators of risk, *n*=29) (bottom).

**Fig. 4.**
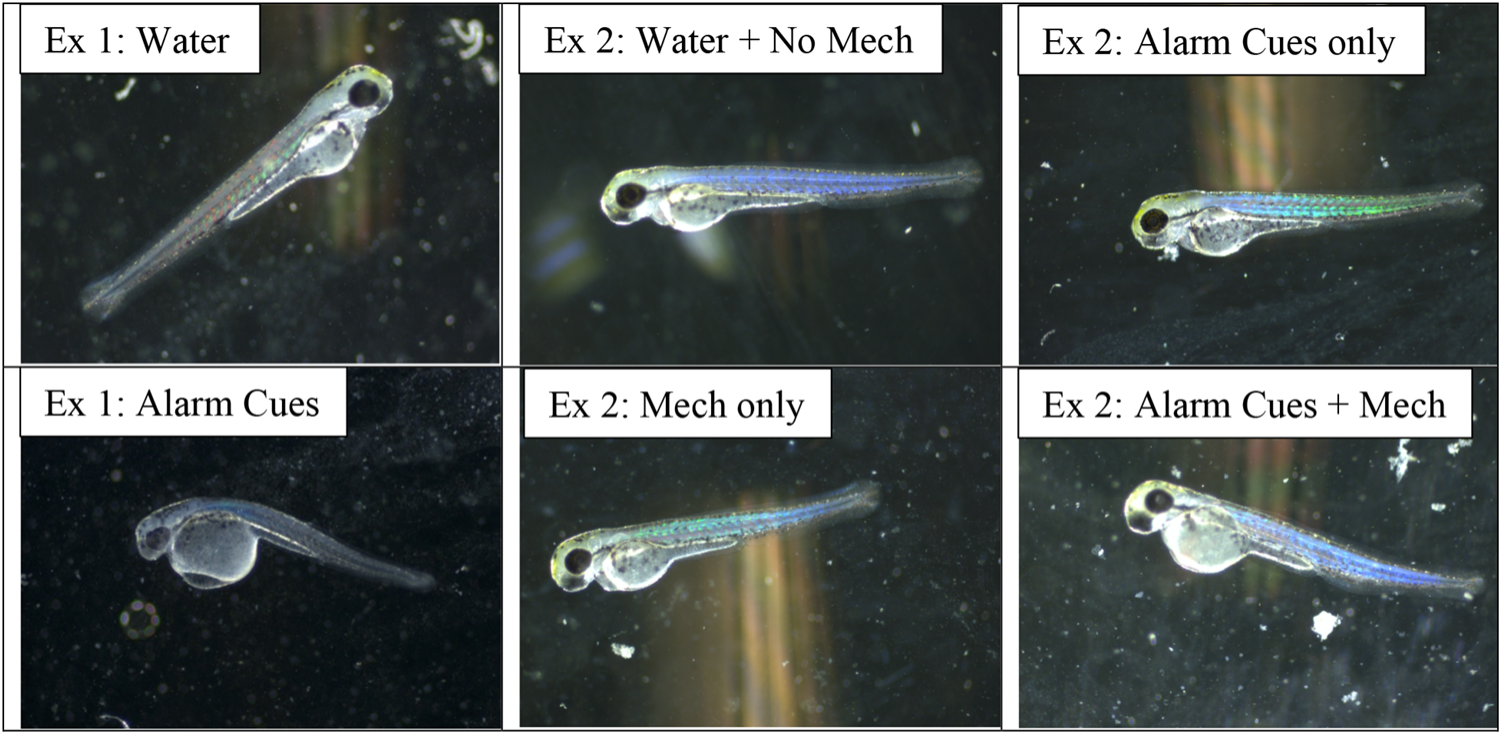
Representative newly hatched zebrafish from the first experiment (Ex 1), comparing the effect of chemical alarm cues versus water, and the second experiment (Ex 2), comparing the independent and combined effects of chemical and mechanical indicators of predation risk.

**Fig. 5.**
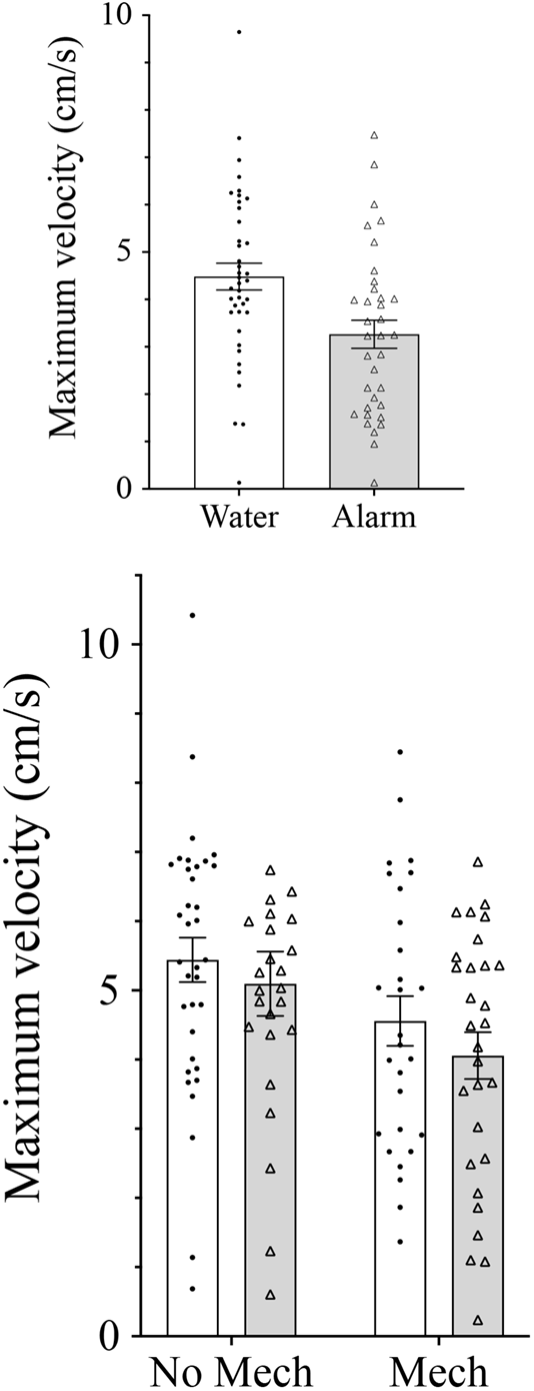
**Effects of chemical alarm and mechanical cues on swimming performance.** In the first experiment, embryos exposed to chemical alarm cues had relatively poor swimming ability (ANOVA *F*_1,45_=7.01, *P=*0.011). In the second experiment, only those embryos exposed to mechanical cues showed poor swimming performance (mechanical cues: ANOVA *F*_1,66_=4.95, *P=*0.030; chemical cues: *F*_1,66_=0.73, *P=*0.396); chemical * mechanical cues: *F*_1,66_=0.02, *P=*0.888). Mean±s.e. maximum velocity (cm/s) of embryos exposed to blank water (open bars, *n*=40) or chemical alarm cues (shaded bars, *n*=35) of conspecific embryos in the first experiment (top), and embryos exposed to chemical and/or mechanical indicators of predation risk in the second experiment (sample sizes: control, *n*=35; mechanical disturbance only, *n*=28; chemical alarm cues only, *n*=25; both chemical and mechanical indicators of risk, *n*=29) (bottom).

**Fig. 6.**
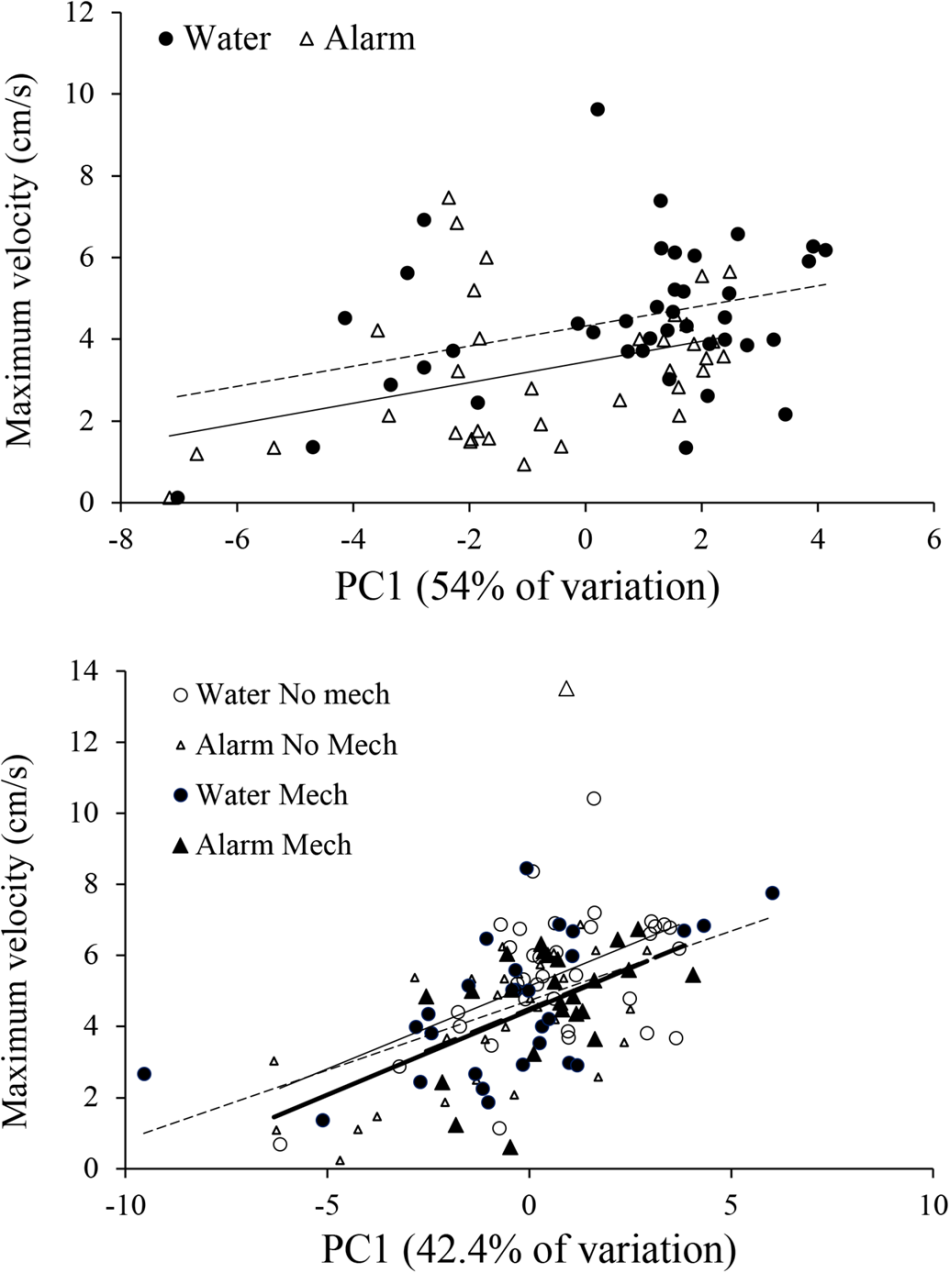
**Effect of hatchling morphology on swimming performance.** Swimming performance was significantly correlated with hatchling morphology (PC1) of embryos exposed to chemical alarm cues in the first experiment (top; ANOVA *F*_1,44_=10.51, *P=*0.002) and from embryos exposed to mechanical cues in the second experiment (bottom; ANOVA *F*_1,65_=45.24, *P*<0.001). Maximum velocity of hatchling larvae as a function of body morphology. Top: larvae incubated as embryos with chemical alarm cues (*n*=35, open triangles, solid line) or water (*n*=40, filled circles, dashed line). Bottom: larvae incubated as embryos with mechanical disturbance (filled symbols, dashed line) or with chemical alarm cues (open and filled triangles, bold lines). Sample sizes: control, *n*=35; mechanical disturbance only, *n*=28; chemical alarm cues only, *n*=25; both chemical and mechanical indicators of risk, *n*=29.

Six of the 15 containers treated with alarm cue and four of the 15 containers treated with blank water had fewer than three larvae hatch, resulting in ten and five missing observations for the alarm cue and control treatments, respectively (effect of treatment was not significant, 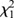
=1.28, *P*=0.258). Treatment with chemical alarm cue resulted in a significantly lower mean hatch time compared to treatment with blank water (*F*_1,45_=6.09, *P=*0.017; Fig. 2). Principal component (PC)1 explained 54.1% of the variation in the morphological data, and in which there was a significant effect of cue treatment (*F*_1,45_=5.34, *P=*0.026; Fig. 3). PC2 explained 10% of the variation in the morphological data and was not a significant predictor of any of the morphological or behavioral responses (*P*>0.05). Caudal fin height, head depth, eye height and a weak trend toward body length differed between the cue treatments (Fig. 4, Table 1). Additionally, treatment with chemical alarm cue resulted in a significantly lower mean maximum swimming velocity (*F*_1,45_=7.01, *P=*0.011; Fig. 5) compared to embryos exposed to blank water. PC1 was highly associated with both hatch time (*F*_1,44_=100.4, *P*<0.001) and maximum velocity (*F*_1,44_=10.51, *P=*0.002; Fig. 6).

**Table 1.**
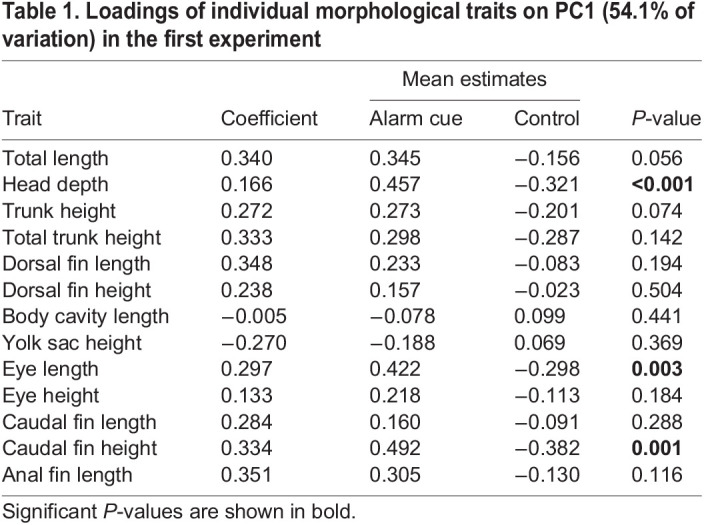
Loadings of individual morphological traits on PC1 (54.1% of variation) in the first experiment

### Experiment #2: Combined effects of chemical and mechanical indicators of predation risk

Each treatment group had between no and two containers with fewer than three larvae hatching, resulting in seven to 17 missing observations per treatment group (no effect of treatment group, 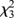
=5.94, *P*=0.115). Treatment with mechanical disturbance was associated with a significantly shorter mean time to hatch compared to treatments with no mechanical disturbance (*F*_1,66_=12.51, *P=*0.001), but there was no effect of chemical alarm cue (*F*_1,66_=0.19, *P=*0.665) or its interaction with mechanical disturbance (*F*_1,66_=0.05, *P=*0.831; Fig. 2). There was a significant effect of mechanical disturbance on PC1 (*F*_1,66_=6.91, *P=*0.011), but no significant effect for chemical alarm cue (*F*_1,66_=0.03, *P=*0.868) or interaction effect (*F*_1,66_=15, *P=*0.697; Fig. 3). PC1 of morphological measurements explained 42.4% of the variation with mechanical disturbance producing hatchlings that were shorter in length, and with relatively poorly developed dorsal, anal and caudal fins (Table 2, Fig. 4). PC2 explained 16% of the variation in the morphological data and was not a significant predictor of any of the morphological or behavioral responses (*P*>0.05). A significantly lower mean maximum swimming velocity was observed for larvae exposed to mechanical disturbance as embryos compared to those that received no mechanical disturbance (*F*_1,66_=4.95, *P=*0.030, Fig. 5). There was no effect of chemical alarm cue (*F*_1,66_=0.73, *P=*0.396) and its interaction with mechanical disturbance (*F*_1,66_=0.02, *P=*0.888) on swimming performance. PC1 was significantly associated with both hatch time (*F*_1,65_=81.28, *P*<0.001) and maximum swimming velocity (*F*_1,65_=45.24, *P*<0.001, Fig. 6).

**Table 2.**
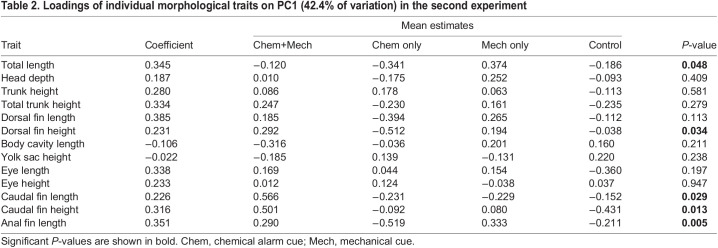
Loadings of individual morphological traits on PC1 (42.4% of variation) in the second experiment

## DISCUSSION

Our data indicate that zebrafish embryos are able to detect and respond to chemical and mechanical indicators of predation risk by accelerating time to hatch. Early hatch would have the effect of reducing exposure to potential egg predators. Embryos that hatched early were underdeveloped and therefore exhibited relatively weak swimming ability. Weak swimming performance would make these early-hatched larvae more vulnerable than control zebrafish to capture by predators of zebrafish larvae (Fuiman and Magurran, 1994). These results concur with previous studies on tadpoles (e.g. Warkentin, 1995) and fathead minnows (Kusch and Chivers, 2004), and add to a growing literature documenting the ability of embryos to adjust time of hatch in response to risk (Warkentin, 2011) with post-hatch fitness consequences. As such, this study adds another example of a trade-off between reduction of risk as embryos by early hatching and an increased risk of predation as newly hatched larvae due to relatively poor swimming performance.

Fish embryos cue hatching to abiotic conditions such as flooding (DiMichele and Taylor, 1980; DiMichele and Westerman, 1997), mechanical cues of returning spring tide (Griem and Martin, 2000), low dissolved oxygen (Czerkie et al., 2011) or risk of desiccation (Wedekind and Müller, 2005). Responses of fish embryos to biological attack are known from only a few studies, in response to pathogens (Warkentin et al., 2001; Pompini et al., 2013) and in response to chemical indicators of risk (Kusch and Chivers, 2004). Our results were mixed. Chemical alarm cues produced a clear effect on hatch time and, consequently, swimming performance in the first experiment, but the second experiment failed to reproduce this effect even though the protocols were identical. We note that the effect of alarm cues on embryos is inconsistent in the literature. Kusch and Chivers (2004) demonstrated that fathead minnows hatch early when exposed to the odor of a crayfish eating minnow eggs, but the effect of chemical cues of crushed embryos alone was not sufficient to induce this effect, nor were alarm cues derived from skin extract from adult fathead minnows (Horn and Chivers, 2021b). Similarly, Crowder and Ward (2022) found no effect of exposure to alarm cues on time to hatch or the length of the hatching interval in fathead minnows, but hatchling larvae from treatments exposed to alarm cues were smaller at 21 days post fertilization. In non-fish taxa, chemical cues from snakes or injured tadpoles did not induce early hatching in red-eyed treefrog tadpoles (Warkentin, 2005). Clearly, additional testing on other species is required before generalizations can be made about the effect of chemical alarm cues on embryonic development. Nevertheless, in at least one of the two replicates of the current study, our data suggest that zebrafish embryos can respond to chemical alarm cues, and mechanical disturbance, with change in time to hatch.

Morphological differences in hatchlings between early-hatched embryos and control embryos, regardless of whether early hatch was induced by chemical alarm cues or mechanical disturbance, showed similar morphological shifts. In both experiments, early hatch was achieved by hatching at an earlier stage of development, as evidenced by shorter body length and relatively underdeveloped dorsal, anal and caudal fins. Thus, our results concur with Kimmel et al. (1995) in that zebrafish have a window of hatchability that allows them to facultatively hatch when embryos detect imminent danger.

Data from the current study showed different hatching times for each iteration of the experiment using the same protocol. There is/are no obvious reason(s) for the variation in hatching times between the two experiments. Extraneous (unmeasured) variables that may influence time to hatch were controlled within each experiment but not between experiments. We also had an unavoidable gap in observation times overnight so that there was no mechanism to distinguish hatches that occurred shortly after the final check of the day and those that hatched just before the first check the following morning. If the peak of hatching events was recorded on one day or the other, mean hours to hatch could appear to shift by the number of hours that differed between our two experiments. Whatever the cause for this variation, the experimental demonstration of the effect of disturbance, either chemical or mechanical, on time to hatch and subsequent swimming performance of hatchlings is unaffected.

The potential for zebrafish embryos to hatch occurs from 48-72 h post fertilization (hpf) at 28.5°C, ranging from the ‘pectoral fin stage’ to the end of the ‘protruding mouth stage’. Kimmel et al. (1995) refer to developing zebrafish as ‘embryos’ until 72 hpf (at 28.5°C) whether they have hatched or not, and embryonic development continues apace whether they have hatched or not because embryos that spontaneously hatch are not more developmentally advanced than ones remaining within the chorion (Kimmel et al., 1995). Timing of exogenous feeding is independent of time of hatching because development continues to be fueled by yolk reserves. During ‘hatching stage’ (48-72 hpf at 28.5°C), zebrafish grow and differentiate their pectoral fins and reinforce them with actinotrichia. This is also when cartilage forms in the head and pectoral fins, the semicircular canals differentiate, circulation and gill tissues develop, and swimming behavior ends in dorsal attitude (Kimmel et al., 1995). Facultative hatching is achieved by proteolytic softening of the chorion (Kim et al., 2004). Body size, fin area and other traits not measured here, such as trunk musculature and skeletal ossification, all contribute to effective escape responses in fish larvae generally (Fuiman and Magurran, 1994; Fuiman et al., 2006; Wisenden et al., 2015; 2016).

Our spawning temperature was cooler, and rearing temperature was warmer, than the standard 28.5°C used by Kimmel et al. (1995); however, the rearing temperature is within the range of temperatures experienced by zebrafish in the natural areas where zebrafish live. The Indian subcontinent and adjacent areas experience temperatures from 16 to 38°C (López-Olmeda and Sánchez-Vázquez, 2011), and because we held all treatment groups in a water bath of constant temperature, our results are attributable to the disturbance cues applied, not temperature.

The evolution of developmental plasticity is an interesting phenomenon in that it occurs when responses are costly, but the benefits occur sporadically over time and space (Warkentin, 2011). Plastic hatching cued to reliable environmental triggers is a sophisticated solution that is superior to polymorphisms or selection toward one morphology or another. Active ingredients in crushed embryos that provide this environmental trigger are not known, nor are the olfactory receptor mechanisms and developmental pathways that are upregulated in response to the switch to early hatching.

Vibrational stimuli that mimic mechanical stimuli of snakes and wasps activate early hatching by red-eyed treefrog tadpoles, whereas mechanical stimuli similar to the mechanical vibrations caused by rain drops, wind or experimental white noise do not (Warkentin, 2005; Caldwell et al., 2009). The duration, frequency and length of intervals between stimuli are important properties that distinguish vibrational stimuli of a snake attack from benign sources of mechanical disturbance (Warkentin, 2005; Warkentin et al., 2006; Caldwell et al., 2009). Specifically, the mechanical stimuli produced by biting and pulling at the egg clutch, done by both snakes and wasps despite their other differences, reliably induce early hatching of tadpole embryos (Caldwell et al., 2009). In the current experiment, the low-level agitation of the incubation container by a small stir bar was not designed to mimic any particular predator species, or mode of predation. Agitation of the substrate where zebrafish embryos develop would occur from general probing behavior of bottom-feeding fishes and invertebrates that likely represent major predators of zebrafish embryos in nature. Spates following a heavy rain event may produce similar agitation of the substrate. Further experimentation to characterize the salient properties of mechanical disturbance that induce early hatching await future experimentation.

These findings open many new avenues for future study because the zebrafish is a model organism for the study of molecular genetics. Environmentally induced hatching in this species provides a convenient model to explore the proximate mechanisms of development and, ultimately, behavioral phenotype of the offspring.

## MATERIALS AND METHODS

Research-grade wild-type zebrafish were purchased from a commercial supplier (EkkWill Water Life Resources, Ruskin, FL, USA). Zebrafish were held in 74-l glass aquaria filtered by external hang-on-back power filters, and fed commercial flake food. Aquaria were filled with dechlorinated tap water heated to 24°C, and the lights were set to a 12:12 h light-dark cycle (as per MSUM IACUC protocol 19-R/T-BIO-018-N-Y-C).

Fertilized zebrafish eggs were collected from colonies of approximately 30 mixed-sex adults housed in 74-l aquaria. Tubs of glass marbles were placed into each of seven separate breeding colonies during darkness approximately 90 min before the lights were due to come on. Zebrafish ovulate overnight, and spawning is most active at dawn (Nasiadka and Clark, 2012). After 2 h, i.e. allowing for 30 min of spawning activity, marble bins were removed from the breeding colonies. Thus, embryos collected each day were uniformly between 0.0 and 0.5 hpf of random mixed parentage. The marbles were then removed from each tub, and the zebrafish embryos were individually collected with a transfer pipette with the tip cut to increase the size of the opening. To make one dosage of chemical alarm cue treatment, ten embryos were transferred into a 1.5-ml centrifuge tube with an additional 1 ml of water from the breeding tanks. We used a microcentrifuge pestle to homogenize embryos in the tube to create a chemical alarm cue. The control cue was prepared by freezing 1-ml doses of tank water in 1.5-ml centrifuge tubes. Cue was frozen at −20°C until needed.

### Experimental protocol

Zebrafish embryos were harvested as described above and arbitrarily assigned to one of six 473-ml containers (flat-bottomed cylinders, 8.0 cm tall, 10.5 cm diameter) filled with 400 ml of dechlorinated tap water. Each container received eight zebrafish embryos. Three containers were assigned to the chemical alarm cue treatment, and three containers were assigned to the water control treatment. The six containers were wedged into holes cut into thin strips of expanded polystyrene that floated in a 74-l aquarium kept at a temperature of 35°C. The relatively high rearing temperature was not planned and not noticed until after all the data were collected. We note that 35°C is within the natural range of in the natural habitat of this species, and because all treatments were reared at a common temperature, temperature is not likely to have had any effect on the effects of experimental treatments. Thus, incubation temperature was kept constant and standardized among all containers and treatment groups. At 11:00 h on day 1 (i.e. approximately 2 hpf), embryos were floated in the incubator tank, placed alternatively to control for positional effects, if any. No additional aeration was provided because agitation caused by an airstone would have confounded the effects of our experiment. Although dissolved oxygen was not measured, the metabolic consumption of oxygen by ten zebrafish embryos, each measuring 0.7 mm in diameter (Kimmel et al., 1995), would not consume oxygen at a rate faster than diffusion at the surface of containers exposed to the open air with 86.6 cm^2^ of surface area and water depth of 5 cm.

Four 1-ml dosages of treatment, via a 1000-μl pipette, were given to each container every day, at 11:00 h, 14:00 h, 18:00 h, and 21:00 h on the first day, and, on subsequent days, treatments were given at 08:00 h, 12:00 h, 16:00 h and 21:00 h. At each of these times, each container was inspected for the presence of hatched larvae. Cue treatments continued to each container until three larvae hatched. When a hatchling larva was observed, date and time were recorded, and the larva was photographed using a ZEISS Stemi 508 microscope with Zeiss Efficient Navigation (ZEN) software. Maximum swimming speed was measured by videotaping each larva responding to a gentle nudge with a pencil tip. We conducted five trials of six containers each, giving a sample size of *n*=15 containers for each treatment group.

ImageJ software was used to take morphological measurements from images of newly-hatched larvae (Fig. 1). We recorded 13 traits: total body length, head depth through the middle of the eye, height of the trunk musculature at the point of the anus, total height of the trunk musculature including the dorsal and anal fin at the point of the anus, height of dorsal fin, length of the dorsal fin, height of the yolk sac, length of the body cavity, eye length, eye height, caudal fin height, caudal fin length, and length of the anal fin from the anus to the tip of the caudal fin. Logger Pro^®^ software was used to calculate the maximum swimming velocity of each larva from video playback.

In the second experiment, we repeated the treatment of chemical alarm cues created from ten crushed embryos per ml, and added an additional treatment of mechanical disturbance in a 2×2 factorial design. The mechanical disturbance was created by placing a small stir bar (13 mm long×8 mm wide) in the container and running a magnetic stirrer for 30 s at medium speed (approximately 1.8 revolutions/s). Trials were run in blocks of multiples of four (one for each treatment combination of chemical cue × mechanical disturbance), i.e. either 12, 16 or 24 tubs in the incubation water bath.

### Statistical analysis

Larvae that did not hatch or that died shortly after hatching were removed from the dataset prior to statistical analysis being performed. The first principal component (PC1) of the 13 morphological measurements was used to obtain an overall index of body form for each fish. In the first experiment, a mixed effects linear model was fit to the data from each outcome, with cue effects considered fixed, and week and container effects considered random. A follow-up analysis was performed on the number of hours until hatch and maximum velocity data by adding the body form index (PC1) as a covariate to the mixed effect models. A similar analysis was performed in the second experiment, but with mechanical disturbance and its interaction with alarm cue added as fixed effects in the model. The mean estimates and standard errors (s.e.) from the mixed effects model analyses were determined. Statistical significance was set at *P*<0.05 based on two-tailed probability distributions. All analysis was performed using the MIXED procedure of SAS software, Version 9. Data are available on Dryad: https://doi.org/10.5061/dryad.zpc866tbg.
